# The leptin sensitizer celastrol reduces age‐associated obesity and modulates behavioral rhythms

**DOI:** 10.1111/acel.12874

**Published:** 2019-03-01

**Authors:** Karthikeyani Chellappa, Isaac J. Perron, Nirinjini Naidoo, Joseph A. Baur

**Affiliations:** ^1^ Department of Physiology and Institute for Diabetes, Obesity and Metabolism Perelman School of Medicine University of Pennsylvania Philadelphia Pennsylvania; ^2^ Center for Sleep and Circadian Neurobiology Perelman School of Medicine University of Pennsylvania Philadelphia Pennsylvania

## Abstract

The prevalence of obesity increases with age in humans and in rodents. Age‐related obesity is characterized by leptin resistance and associated with heightened risk of metabolic disorders. However, the effect of leptin resistance per se has been difficult to disentangle from other effects of aging. Here we demonstrate that celastrol, a natural phytochemical that was previously shown to act as a leptin sensitizer, induces weight loss in aged animals, but not in young controls. Celastrol reduces food intake and lowers fasting glucose without affecting energy expenditure. Unexpectedly, administration of celastrol just before the dark period disrupted circadian rhythms of sleep and activity. This regimen was also associated with loss of lean mass an outcome that would not be desirable in elderly patients. Adjusting the timing of celastrol administration by 12 hr, to the beginning of the light period, avoided interference with circadian rhythms while retaining the reductions in body weight and adiposity. Thus, targeting leptin signaling is an effective strategy to ameliorate age‐associated weight gain, and can profoundly impact circadian rhythms.

## 1 INTRODUCTION

The aging population is rising worldwide, with aged individuals 65 years or older projected to represent more than 20% of the population in the United States by 2035 (Mathus‐Vliegen, 2012). Obesity becomes increasingly more prevalent with age, and is a major risk factor for many conditions including cardiovascular disease, hypertension, stroke, diabetes, dyslipidemia, cognitive decline, and mortality (Chung, Kang, Lee, Lee & Lee, 2013; Dominguez & Barbagallo, 2016; Han & Lean, 2016; Saag & Choi, 2006). In young individuals, the hormone leptin plays a key role in maintaining energy balance and body weight. Leptin is secreted by adipose tissue in proportion to its mass to relay information on peripheral energy stores to the central nervous system (Ahima, Saper, Flier & Elmquist, 2000; Frederich et al., 1995). Binding of leptin to its receptor expressed in different sites within the brain can suppress food intake and increase energy expenditure, thus serving as a negative feedback on energy storage (Ovesjö, Gamstedt, Collin & Meister, 2001; Balthasar et al., 2004; Scott, Williams, Rossi, Lee & Elmquist, 2011; Billes, Simonds & Cowley, 2012; Rezai‐Zadeh et al., 2014; Li, Kelly, Heiman, Greengard & Friedman 2015). Aging in humans and rodents is characterized by an expansion of adipose mass in middle age that is not resolved, despite increased circulating leptin levels (Justesen et al., 2001; Kotani et al., 1994; Kuk, Saunders, Davidson & Ross, 2009; Kyle et al., 2001; Muzumdar et al., 2008; Shek & Scarpace, 2000; Visser et al., 2003). The inability of elevated leptin to bring about appropriate metabolic and endocrine outcomes, including the decrease in food intake and body weight, is termed leptin resistance (Carter, Caron, Richard & Picard, 2013; Gabriely, Xiao Hui, Yang, Rossetti & Barzilai, 2002; Ma et al., 2002; Myers et al., 2012; Pétervári et al., 2014). The underlying mechanism of leptin resistance with age is debated, and has alternately been attributed to disruption of leptin transport across the blood‐brain barrier, decreased leptin receptor expression, feedback inhibition of leptin signaling by downstream effectors such as SOCS3 and TCPTP, or chronic ER stress and inflammation (Bigford, Bracchi‐Ricard, Nash & Bethea, 2012; Fernández‐Galaz et al., 2001, 2002; González‐Rodríguez et al., 2012; Martínez, Duran‐Aniotz, Cabral‐Miranda, Vivar & Hetz, 2017; Peralta, Carrascosa, Gallardo, Ros & Arribas, 2002; Rostás et al., 2016; Scarpace, Matheny & Tümer, 2001). Although improved hypothalamic leptin signaling correlates with the amelioration of age‐associated obesity in several models (Fernández‐Galaz et al., 2002; Sasaki et al., 2014; Yang et al., 2012), the contribution of leptin resistance per se has been difficult to ascertain.

Celastrol, a phytochemical isolated from the thunder god vine (Tripterygium Wilfordi) was recently identified as a leptin sensitizer based on its ability to suppress food intake and reduce body weight in diet‐induced obese mice, but not in lean mice or in obese mice with genetically disrupted leptin signaling (Liu, Lee, Hernandez, Mazitschek & Ozcan, 2015). Thus, its effects are contingent upon elevated plasma leptin levels and leptin receptor expression. We hypothesized that celastrol might also be an effective strategy to restore leptin sensitivity and body weight homeostasis in aged mice, which, like obese mice, display hyperleptinemia and leptin resistance. We report that celastrol ameliorates leptin resistance in aged mice and decreases body weight, but unexpectedly had adverse effects on the circadian rhythms of locomotor activity and sleep when administered prior to the lights off active period. Switching the time of drug delivery to just before the onset of the light period avoided the circadian effects while still maintaining the reductions in food intake and body weight. Therefore, celastrol is effective in treating age‐related obesity, but the time of delivery has a profound impact on the outcome of treatment.

## 2 RESULTS

### 2.1 Celastrol ameliorates age‐associated leptin resistance

**Figure 1:**
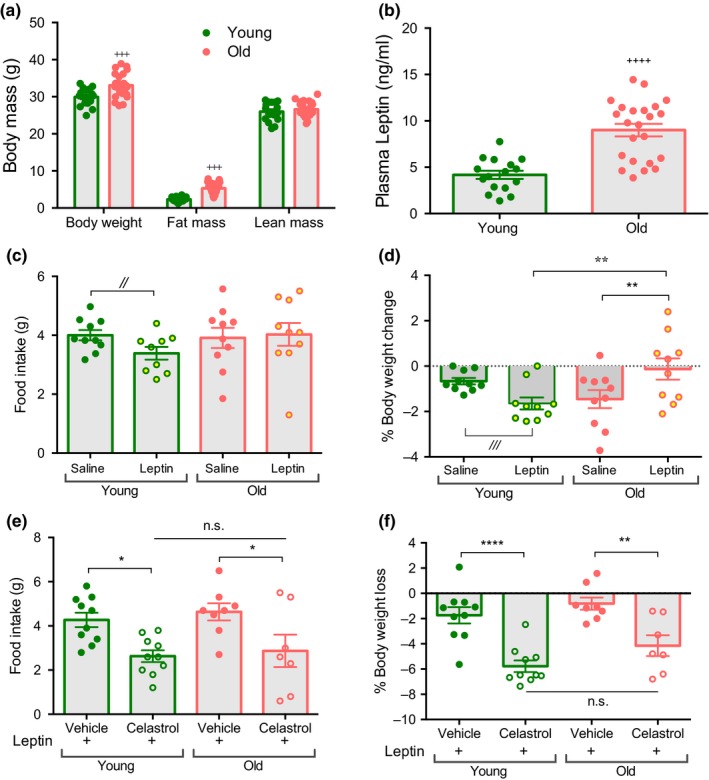
Celastrol restores leptin sensitivity in aged mice. (a) Body composition of young (4 month) and old (18 month) male mice. *n* = 16–23. (b) Plasma leptin of young (4 month) and old (18 month) mice. *n* = 16–23. (c) 24 hr food intake measurement during saline (average of 5 d) and leptin injection in young (4 month) and old (21 month) mice. *n* = 9–10. (d) % body weight change during saline (average of 5 d) and leptin injection in young (5 month) and old mice (21 month). *n* = 9–10. (e) Food intake of vehicle or celastrol treated mice after 24 hr of leptin injection in young (6 month) and old (22 month) mice. *n* = 7–10. (f) % Body weight change of vehicle or celastrol treated mice after 24 hr of leptin injection in young (6 month) and old (22 month) mice. *n* = 7–10. All data are presented as mean ± *SEM*s. *p < 0.05, **p < 0.01, ^+++^ *p *< 0.005, ^++++^ *p *< 0.0001, ^//,///^indicated comparisons are non‐significant when corrected for multiple comparisons (ANOVA) but are nominally significant by Student's *t*‐test (^//^ *p *< 0.05, ^///^ *p *< 0.005)

Aged mice show an increase in body weight and fat mass without any change in lean mass (Figure 1a), as previously reported (Houtkooper et al., 2011). Consistent with the increased adipose mass, aged mice display higher plasma leptin concentrations (Figure 1b). A single injection of recombinant leptin tended to decrease body weight and food intake, in young but not in aged mice (Figure 1c,d), consistent with previous reports that aged mice are leptin resistant (Fernández‐Galaz et al., 2002; Gabriely et al., 2002). Next, we determined the ability of celastrol to reestablish leptin signaling in aged mice. We found that 2 days of celastrol pre‐treatment sensitized aged mice to leptin's action on food intake and body weight (Figure 1e,f). Importantly, the difference in leptin sensitivity between young and aged mice was completely abolished after celastrol treatment.

### 2.2 Celastrol decreases body weight in aged mice by reducing food intake

**Figure 2:**
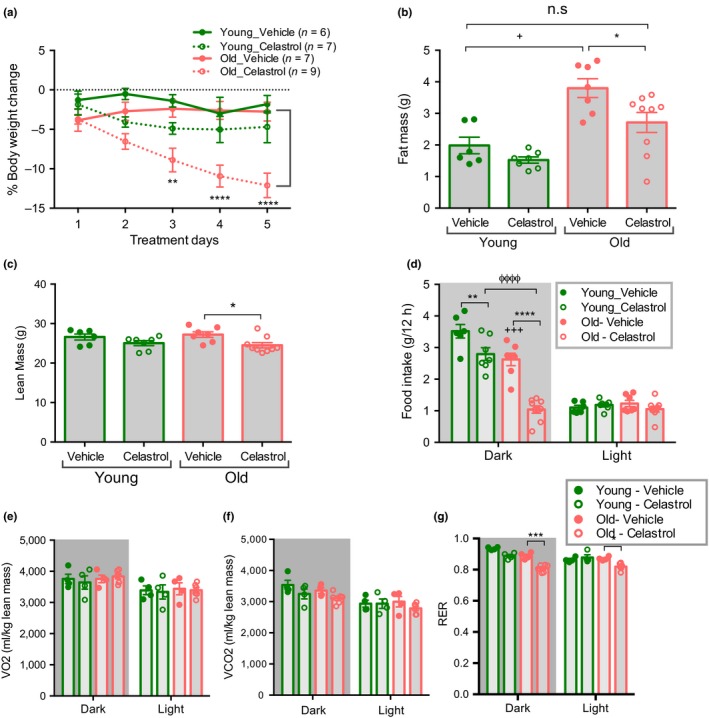
Celastrol decreases food intake and body weight in aged mice. (a) % Body weight change in 4 month young and 18–20 month aged mice treated with either vehicle or celastrol. *n* = 6–9. (b) Fat mass of mice treated with vehicle or celastrol for 5 d as in (a). *n* = 6–9. (c) Lean mass of mice treated with vehicle or celastrol for 5 d as in (a). *n* = 6–9. (d) Average food intake during 5 days of vehicle or celastrol treatment as in (a). *n* = 6–9. (e) Average VO2 normalized to lean mass during 5 days of vehicle or celastrol treatment as in (a). *n* = 4–6. (f) Average VCO2 normalized to lean mass during 5 days of vehicle or celastrol treatment as in (a). *n* = 4–6. (g) Average RER during 5 days of vehicle or celastrol treatment as in (a). *n* = 4–6. All data are presented as mean ± *SEM*s. *p < 0.05, **p < 0.01, ***p < 0.005, ****p < 0.0001, ANOVA comparison between vehicle and celastrol treated group; ^+^ *p *< 0.05, ^+++^ *p *< 0.005, ^++++^ *p *< 0.0001 ANOVA between vehicle treated young and old mice, ^ϕϕϕϕ^ *p *< 0.0001 ANOVA between celastrol treated young and old mice

To assess the effect of celastrol on body weight homeostasis young and aged mice were intraperitoneally injected with vehicle or celastrol for 5 days. We found that aged mice lost ~11.5% of their initial body weight with celastrol treatment (Figure 2a). Celastrol treatment decreased both fat mass and lean mass in aged mice (Figure 2b,c). On the other hand, celastrol did not cause a significant change in body weight, fat or lean mass in young mice, as previously reported (Liu, Lee, Hernandez, Mazitschek, Ozcan, 2015). This difference is most likely attributable to the lower circulating leptin concentrations in young, leptin sensitive animals. Celastrol lowered fasting glucose in both young and old mice and reduced the absolute area under the glucose curve during an insulin tolerance test (ITT) in aged mice (Supporting information Figure S1a‐b). However, the effect of celastrol on ITT is no longer significant when the data are plotted as percent change (Supporting information Figure S1c‐d), suggesting that celastrol may in part lower glucose through mechanisms independent of insulin sensitivity per se. Celastrol treatment significantly decreased food intake during the dark period, with a much more pronounced effect in aged mice (Figure 2d). Food intake correlates with water intake in both lean and obese rodents (Fitzsimons & Le Magnen, 1969; Strominger, 1947). Consistently, we found that celastrol reduced water consumption in aged mice more than in young mice (Supporting information Figure S2a). In contrast, energy expenditure was unaffected by celastrol treatment in both age groups (Figure 2e,f). RER was decreased only in celastrol treated aged mice, consistent with reduced food intake (Figure 2g). Thus, our data establish that a decrease in food intake in the absence of any overt change in energy expenditure leads to body weight loss in celastrol treated old mice.

### 2.3 Celastrol treatment disrupts circadian rhythms of locomotor activity and sleep

**Figure 3:**
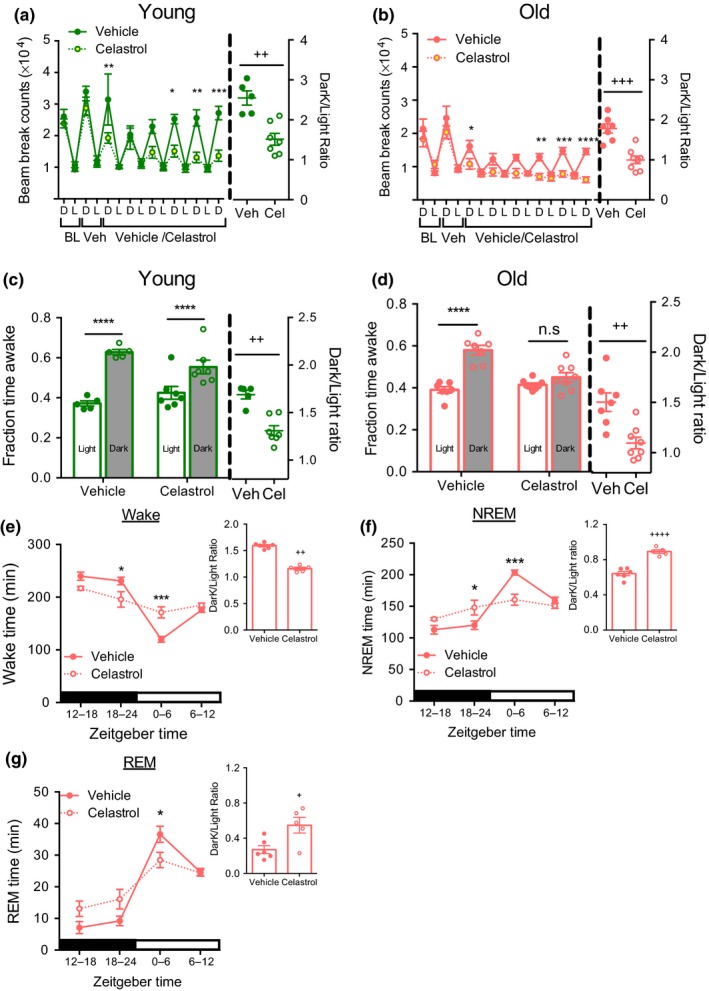
Celastrol treatment disrupts circadian activity and sleep pattern. (a) Beam break counts (left) and dark to light counts ratio (right) of young mice treated with vehicle or celastrol as in Figure 2a. *n* = 5–7. (b) Beam break counts (left) and dark to light counts ratio (right) of old mice treated with vehicle or celastrol as in Figure 2a. *n* = 7–8. (c) Fraction wake time (left) and dark:light ratio of young mice in (a) determined using beam break counts as in Figure 2a. *n* = 5–7. (d) Fraction wake time (left) and dark:light ratio of old mice as treated in Figure 2a determined by beam break counts. *n* = 7–8. (e) Wake time (left) and dark light ratio (right) as recorded by EEG in aged (20 month) mice treated with cealstrol for Figure 4d. *n* = 5–6. (f) NREM sleep (left) and dark light ratio (right) measured as in (d). *n *= 5–6. (g) REM sleep (left) and dark light ratio (right) recorded as in (d). *n* = 5–6. All data are presented as mean±*SEMs*. *p < 0.05, **p < 0.01, ***p < 0.005, ****p < 0.0001, ANOVA comparison between vehicle and celastrol treated groups; ^+^ *p *< 0.05, ^++^ *p *< 0.005, ^+++^ *p *< 0.001 and ^++++^ *p *< 0.0001, unpaired *t*‐test between vehicle and celastrol treatment

Locomotor activity, sleep/wake architecture and circadian rhythms decline with age (Houtkooper et al., 2011; Naidoo et al., 2011; Wimmer et al., 2013). Accordingly, we observed reduced locomotor activity in aged mice (Figure 3a,b). Celastrol treatment significantly decreased total locomotor activity in both young and aged mice.

Strikingly, the circadian rhythm of activity was significantly altered after celastrol treatment in both age groups. Liu et al. (2015) also reported decrease in locomotor activity in young lean and diet‐ induced obese mice treated with celastrol, and suggested that this might reflect reduced food‐ seeking behavior. Given that we and others have shown that locomotor activity can predict sleep patterns (Brown, Hasan, Foster & Peirson, 2017; Pack et al., 2007), we chose to examine the alternative hypothesis that celastrol affects the circadian timing of sleep. Specifically, we have previously established that a period of immobility as estimated by lack of beam break for >40 s is an accurate predictor of sleep (Pack et al., 2007). We used this parameter to determine total sleep and wake time, and estimated diurnal ratio to measure changes in circadian rhythm. Based on this analysis, the vehicle‐treated young and aged mice displayed increased wake time during the active dark period compared to the inactive light period (Figure 3c,d). Paralleling the effect on activity, the circadian pattern of sleep/wake cycles was disrupted in mice treated with celastrol in both of the age groups. Celastrol had more pronounced effects in aged mice, resulting in complete loss of the circadian pattern of wake time. To formally confirm the effect of celastrol on sleep we next performed electroencephalography (EEG) measurements in aged mice. Reduced body weight and food intake were observed in response to celastrol after EEG instrumentation as in non‐instrumented mice (Supporting information Figure S2b‐c). EEG measurements confirmed that celastrol decreased wake time during the dark period while increasing it during light period, resulting in a flattening of circadian rhythms of sleep (Figure 3e). Furthermore, the decrease in wakefulness in the dark period paralleled an increase in both total NREM sleep and REM bouts (Figure 3F,G and Table 1).

**Table 1:** Electroencephalography (EEG) recording data of aged mice treated with vehicle or celastrol

	Vehicle	Celastrol	Significance
	Mean **± ** *SEM*	Mean ± *SEM*	
Wake			
Number of bouts			
24‐hr	446.5 ± 21.9	437.2 ± 11.2	ns
Light	230.8 ± 15.0	211.6 ± 15.6	ns
Dark	215.7 ± 14.4	225.6 ± 9.2	ns
Bout length			
24‐hr	104.1 ± 4.9	105.5 ± 2.6	ns
Light	78.32 ± 4.6	103.7 ± 10.2	ns (*p* < 0.086)
Dark	134.3 ± 10.8	110.3 ± 5.2	ns (*p* < 0.1)
NREM			
Number of bouts			
24‐hr	447.5 ± 22.0	437.4 ± 10.8	ns
Light	231.3 ± 14.7	212.0 ± 15.4	ns
Dark	216.2 ± 14.7	225.4 ± 8.9	ns
Bout length			
24‐hr	80.9 ± 4.1	81.2 ± 3.9	ns
Light	96.1 ± 6.1	90.2 ± 7.4	ns
Dark	65.6 ± 3.5	74.6 ± 4.8	ns
REM			
Number of bouts			
24‐hr	89.2 ± 7.7	106.2 ± 12.8	ns
Light	69.8 ± 5.4	63.2 ± 5.3	ns
Dark	19.3 ± 4.2	43.0 ± 8.8	a
Bout length			
24‐hr	53.1 ± 2.4	47.2 ± 2.5	ns
Light	53.4 ± 2.6	50.8 ± 2.2	ns
Dark	54.5 ± 4.0	41.9 ± 3.1	ns (*p* < 0.1)

a *p* < 0.05, repeated measures two way ANOVA following Sidak post‐hoc test.

Taken together, our finding suggests that celastrol adversely affects circadian rhythms of activity and sleep.

### 2.4 Celastrol delivery at ZT23 abrogates age‐associated obesity

**Figure 4:**
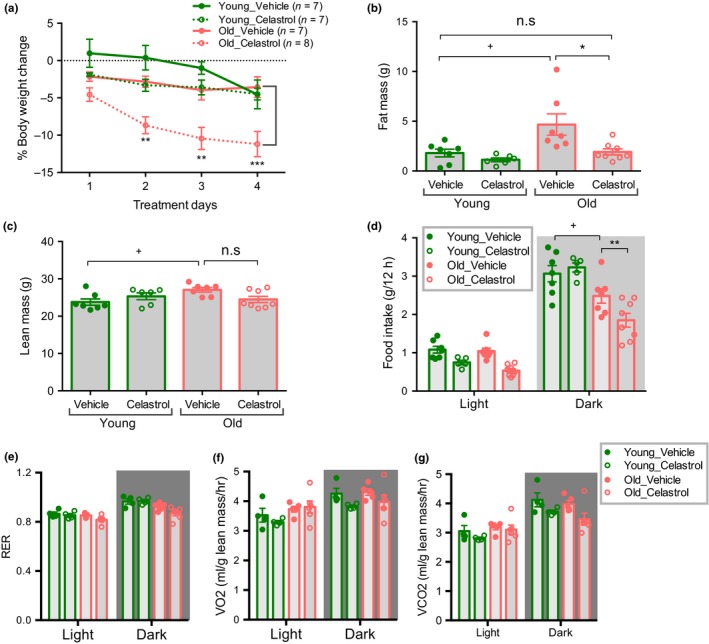
Celastrol delivery at ZT23 ameliorates age‐associated obesity. (a) % Body weight change in 4 month young and 18–20 month old mice treated with either vehicle or celastrol. *n *= 6–8. (b) Fat mass of mice treated with vehicle or celastrol for 4 d as in (a). *n* = 6–8. (c) Lean mass of mice treated with vehicle or celastrol for 4 d as in (a). *n* = 6–8. (d) Average food intake during 4 days of vehicle or celastrol treatment as in (a). *n =* 5–8. (e) Average VO2 normalized to lean mass during 4 days of vehicle or celastrol treatment as in (a). *n* = 4–6. (f) Average VCO2 normalized to lean mass during 4 days of vehicle or celastrol treatment as in (a). *n* = 4–6. (g) Average RER during 4 days of vehicle or celastrol treatment as in (a). *n *= 4–6. All data are presented as mean ± *SEM*s. *p < 0.05, **p < 0.01, ***p < 0.001, ANOVA comparison between vehicle and celastrol treated groups; ^+^ *p *< 0.05, ANOVA between vehicle treated young and aged mice

Our results thus far show that celastrol negatively influences sleep and wake, by decreasing wake time during their active period and increasing wake time during their inactive period (Figure 3). We reasoned that if celastrol provided a circadian cue, then shifting the time of delivery might reverse its effect on circadian rhythms. Notably, a circadian rhythm has been described for plasma leptin, peaking before the onset of the light period and falling over the course of the day (Ahima, Prabakaran & Flier, 1998; Sukumaran, Jusko, DuBois & Almon, 2011). Injection at ZT23 (before the onset of the light period and 12 hr later than in the previous study) decreased body weight and adipose mass in aged mice (Figure 4a,b), similar to the effect at ZT11 injection. In contrast to ZT11 injection, ZT23 injection did not significantly decrease lean mass in aged mice (Figure 4c). Indirect calorimetry confirmed that the weight loss induced by ZT23 injection was primarily driven by a decrease in food intake and RER without any change in energy expenditure in aged mice (Figure 4d‐g, Supporting information Figure S2d).

### 2.5 Celastrol delivery at ZT23 restores circadian rhythms of activity and sleep

**Figure 5:**
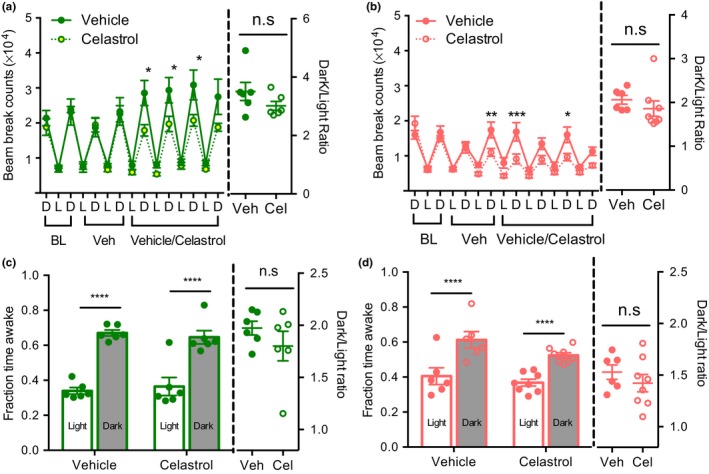
Celastrol injection at ZT23 restores circadian pattern of behavior. (a) Beam break counts (left) and dark to light counts ratio (right) of 4 month young mice treated with vehicle or celastrol. *n* = 6. (b) Beam break counts (left) and dark to light counts ratio (right) of 18–20 month old mice treated with vehicle or celastrol. *n* = 6–8. (c) Average fraction wake time (left) and dark:light ratio determined by beam break counts of 4 month young mice during 4 days of vehicle or celastrol treatment as in (a). *n* = 6. (d) Average fraction wake time (left) and dark:light ratio determined by beam break counts of 18–20 month old mice during 4 days of vehicle or celastrol treatment as in (b). *n* = 6–8. All data are presented as mean ± *SEM*s. *p < 0.05, **p < 0.01, ***p < 0.001, ****p < 0.0001, ANOVA comparison of vehicle and celastrol treated groups; *p *> 0.05 non‐significant (n.s.), unpaired *t*‐test between vehicle and celastrol treatment

To test whether the shift in circadian timing of celastrol delivery could ameliorate its effects on sleep and activity patterns, we performed beam break analysis. Mice injected with celastrol at ZT23 retained the pattern of increased activity during the dark period as compared to the light period similar to vehicle‐treated controls (Figure 5a,b), although a modest reduction in activity during the dark period was still detected. Calculating wake epochs demonstrates that the circadian pattern of the wake period was preserved by ZT23 injection (Figure 5c,d).

Collectively, our data demonstrates that the circadian timing of celastrol delivery can have a profound effect on the circadian rhythms of activity and sleep.

## 3 DISCUSSION

Aging is characterized by an increase in obesity and disruption of metabolic homeostasis, driving intense interest in identifying small molecules that can reverse these effects. The naturally occurring phytochemical celastrol was recently reported to cause weight loss in diet‐ induced obese mice by restoring leptin sensitivity and thereby allowing hyperleptinemia to appropriately suppress food intake. In contrast, the body weights of lean mice with lower leptin levels were not affected. In this study, we determined that age‐related obesity, which involves milder leptin resistance and hyperleptinemia, is also treatable with celastrol. We report that celastrol restores leptin sensitivity, suppresses food intake and lowers body weight selectively in aged mice. Unexpectedly, we found that celastrol injection at ZT11 disrupts the circadian rhythms of locomotor activity and sleep, and that this can be avoided by shifting the timing of administration by 12 hours to ZT23. Thus, celastrol holds promise in the treatment of age‐ related obesity, but requires careful attention to circadian timing of treatment.

We found that intraperitoneal injection of celastrol (200 μg/kg/day) decreases body weight almost exclusively by suppressing food intake in aged mice. Our findings are consistent with previous reports on celastrol‐induced weight loss in DIO mice receiving a similar dosing regimen (Hu et al., 2017; Liu et al., 2015; Zhang et al., 2017). Central leptin signaling was shown to mediate the effect of celastrol on food intake (Liu et al., 2015).

Although the mechanism connecting celastrol to leptin signaling remains elusive, Hu et al., (2017) recently demonstrated that celastrol can bind Nurr77 and induce interaction with TRAF2 to stimulate autophagy and reduce inflammation. They further demonstrate that Nurr77 is required for celastrol‐induced weight loss in vivo and speculate that improved leptin sensitivity might be a consequence of reduced hypothalamic inflammation. Interestingly, celastrol administration at higher doses in the diet (1–3 mg/kg/day) was reported to increase energy expenditure by stimulating thermogenesis and oxygen consumption in adipose tissue and skeletal muscle (Ma et al., 2015). This effect was dependent on an HSF1‐PGC1α pathway that was found to act independently from food intake (Ma et al., 2015). Together, these studies suggest that celastrol treatment can influence different aspects of energy balance to alleviate obesity, depending on the dose and/or route of delivery. It will be interesting in future studies to determine how aging and celastrol treatment affect expression of leptin and its receptor, as well as downstream signaling in the hypothalamus versus peripheral tissues.

We and others have previously shown decreased locomotor activity and fragmented sleep‐wake architecture in aged mice (Houtkooper et al., 2011; Naidoo et al., 2011; Wimmer et al., 2013). Nevertheless, aged mice maintain a higher level of activity during the active dark period compared to the inactive light period. Similarly, aged mice exhibit longer wake time in the dark period, and sleep time during the light period. Our study suggests that administering celastrol just before the dark period disrupts circadian patterns of activity and sleep in both young and aged mice. However, we were able to overcome this effect by switching the time of injection to the light period. We do not believe that celastrol is acting primarily as a somnogen because of the delayed onset of the sleep/wake differences (Figure 3). Interestingly, this fits with the known circadian rhythm of leptin concentration, which peaks during late dark phase (Ahima et al., 1998; Sukumaran et al., 2011). We hypothesize that sensitizing to leptin at the time of its natural peak may be less disruptive to circadian rhythms, and that further optimization of the timing might even strengthen them, though this idea remains to be formally tested. It also remains possible that the effects of celastrol on circadian rhythms could be independent from leptin. The importance of delivering drugs at the optimum time within 24 hr biological rhythms to maximize their efficacy and tolerability is well recognized, specifically in the field of cancer therapeutics (Ballesta, Innominato, Dallmann, Rand & Lévi, 2017; Lévi & Okyar, 2011). Our study reemphasizes the need to assess optimal drug delivery time of celastrol (and possibly leptin) in clinical trials to avoid adverse side effects.

Celastrol, the most potent bioactive material in *Tripterygium Wilfordi* has been extensively tested in the treatment of cancer, lupus, amyotrophic lateral sclerosis, and Alzheimer's disease in rodent models (Cascão, Fonseca & Moita, 2017). However, the clinical relevance of celastrol is currently restricted by insolubility, bioavailability and narrow therapeutic window. Thus, further studies are required to completely understand the wide range of physiological and behavioral effects of celastrol before this compound reaches human therapy. In summary, we demonstrate that celastrol improves leptin sensitivity and ameliorates age‐associated obesity in mice. We further establish that the circadian timing of celastrol delivery impacts the behavioral rhythms of activity and sleep.

## 4 MATERIALS AND METHODS

### 4.1 Animal use and care

Animal experiments were conducted in accordance with guidelines of University of Pennsylvania Institutional Animal Care and Use Committee. Mice were maintained under 12‐hr light/dark cycles at ~21°C and either fed a standard lab chow (Rodent Diet 5010, LabDiet). Young (4–6 month) and Old (18–22 month) male mice were obtained from National Institute of Aging. Mice were sacrificed by cervical dislocation and tissues were harvested and frozen in liquid nitrogen and stored at −80°C until use.

### 4.2 Celastrol administration

Experiments were conducted in either in home cage or metabolic cage. Mice were acclimatized to single housing and vehicle (0.6% DMSO in 10% captisol) injection for 1–4 days and then intraperitoneally injected with vehicle or Celastrol (100–200 μg/kg body weight) for 4–6 days. Injections were carried out either before the onset of dark period at ZT11 (Figure 1, 2, 3 and Supporting information Figure S1) or onset of light period at ZT23 (Figures 4 and 5). Body weight and food intake were recorded daily at the time of injection.

### 4.3 Leptin sensitivity assay

Mice were singly housed in their home cage and intraperitoneally injected with saline at ZT11 for 5 days and monitored for body weight and food intake. On day 6 mice were intraperitoneally injected with leptin (4 mg/kg body weight). For leptin sensitivity after celastrol treatment, mice were injected with either vehicle or celastrol (200 μg/kg body weight) for 2 days. On the 3rd day mice were injected with either vehicle or celastrol 1 hr before leptin injection (5 mg/kg body weight). Body weight and food intake were manually tracked 24 hr after injection.

### 4.4 Metabolic studies

Energy balance after celastrol treatment was monitored using comprehensive lab animal monitoring system (CLAMS) (Columbus Instruments, Columbus, OH). Mice were acclimatized to metabolic cage (1–2 d) and vehicle injection (1–2 d) and then injected with either vehicle or celastrol. Food intake, energy expenditure, RER and activity is presented as an average of 4 d during vehicle or celastrol treatment.

### 4.5 Body composition

Body composition was measured by nuclear magnetic resonance imaging (EchoMRI, Echo Medical Systems, Houston, USA).

### 4.6 Insulin tolerance test

For insulin tolerance test mice were fasted for 6 hr and injected intraperitoneally with human insulin (0.75 IU/kg body weight). Blood glucose was measured periodically using one touch glucometer for 2 hr following injection.

### 4.7 EEG sleep recording

Sleep recordings were conducted as previously described (Perron, Pack & Veasey, 2015). Briefly, silver wire electrodes were soldered to gold sockets and push fit into 6‐channel plastic holders. Mice were anesthsized under isoflurane, and holes were drilled in frontoparietal areas where electrodes were inserted; EMG reference electodes were placed in nuchal muscles. After 1 week of recovery, mice were acclimated to tether cables for 1 week prior to any sleep/wake recordings. Data was acquired with Neurodata amplifier systems, exported to .edf files, and scored manually by an experience investigator blinded to experimental condition. Sleep/wake recordings were measured on Day 0 (prior to celestral treatment) and Day 4 of daily celestral injections. Total amount of wake, NREM sleep, and REM sleep, as well as sleep/wake fragmentation analysis, was calculated in MATLAB.

### 4.8 Statistical analysis

Data are expressed as mean ± *SEM*, of sample size *n*. One‐way or two‐way ANOVA was used with Tukey's or Sidak post‐hoc test for comparisons of three or more groups. Student's *t*‐test was used for two group comparisons or to determine nominal significance. *p *< 0.05 was considered to be significant.
