# Supplementary material for: The leptin sensitizer celastrol reduces age‐associated obesity and modulates behavioral rhythms
Source: Aging Cell. 2019 Mar 1;18(3):e12874. doi: 10.1111/acel.12874 (PMC6516176; doi:10.1111/acel.12874)

Supplementary Figure 1. Effect of celastrol on glucose homeostasis.

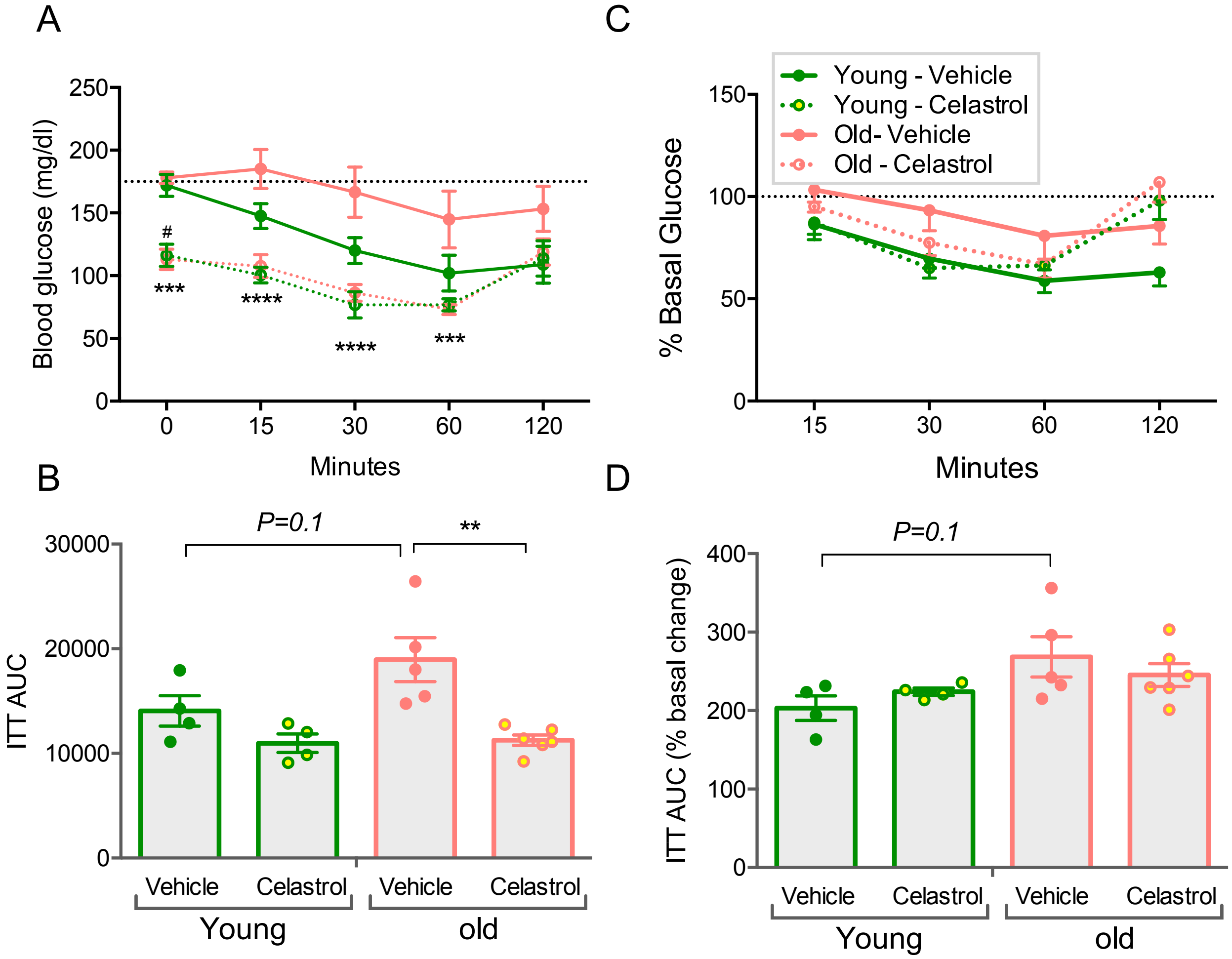

Supplementary Figure 2. Effect of celastrol on food and water consumption

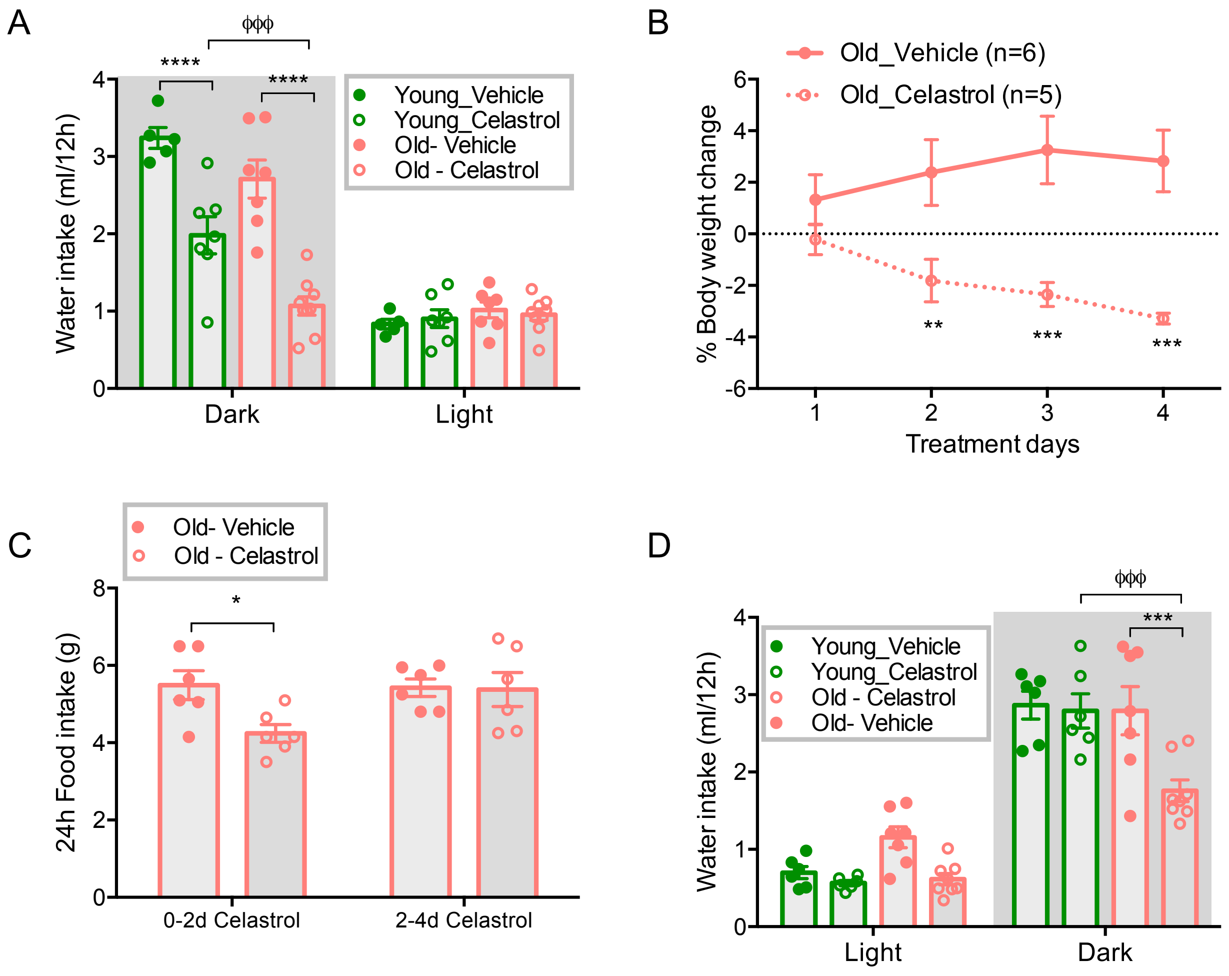

Supplement: Supplementary file 1 [file ACEL-18-e12874-s001.pdf]
